# Minimally invasive anterolateral approach versus direct anterior approach total hip arthroplasty in the supine position: a prospective study based on early postoperative outcomes

**DOI:** 10.1186/s13018-022-03126-0

**Published:** 2022-04-12

**Authors:** Hongwen Liu, Li Yin, Jiao Li, Shaojiang Liu, Qifeng Tao, Jie Xu

**Affiliations:** 1 Department of Orthopaedics, Panzhihua Central Hospital, #34 Yikang Road, Panzhihua, 617067 Sichuan Province China; 2 Department of Discipline Construction Office, Panzhihua Central Hospital, #34 Yikang Road, Panzhihua, 617067 Sichuan Province China; 3 Department of Orthopedics, Fujian Provincial Hospital, Fujian Medical University, #134 East Road, Fuzhou, 350001 Fujian Province China

**Keywords:** Total hip arthroplasty, Minimally anterolateral approach, Direct anterior approach, Early results

## Abstract

**Background:**

Minimally anterolateral approach (MAA) and direct anterior approach (DAA) have been reported as beneficial for total hip arthroplasty (THA) due to their ability to reduce postoperative pain and lead to quicker rehabilitation by preserving muscle insertions. As there is an ongoing debate on the effect of these two approaches on early postoperative outcomes, this prospective study aimed to assess the difference in early clinical, radiological, and patient-reported outcomes between the two minimally invasive approaches.

**Methods:**

A total of 98 patients, 50 in the MAA group and 48 in the DAA group, were included in the study. Patients with complete data were evaluated preoperatively and postoperatively at 2, 6, and 12 weeks. Clinical measurements, including the ability to climb stairs and walk, 6-min walk test (6MWT), the Forgotten Joint Scale (FJS-12), Japanese Orthopedic Association (JOA) Hip scores, radiological evaluation, and complications were analyzed.

**Results:**

There were no significant differences in clinical outcomes and implant alignments between MAA and DAA groups. In regards to patient-reported outcomes, the FJS-12 was significantly higher in the MAA group compared to group DAA at 2 and 6 weeks postoperatively. However, there was no significant difference in the FJS-12 between the two groups 12 weeks after surgery. The differences also included shorter operative times (62.4 ± 9.05 min vs. 71 ± 8.01 min), less blood loss (132.6 ± 43.31 ml vs. 159.23 ± 37.25 ml), lower Hb drop (29.56 ± 8.02 g/L vs. 36.4 ± 7.12 g/L), and fewer blood transfusions in the MAA group (4.0% vs. 18.8%). The incidence of the lateral femoral cutaneous nerve (LFCN) neuropraxia after surgery was 7 (14.6%) in the DAA group and 0 in the MAA group. One fracture was found in each group and managed conservatively.

**Conclusion:**

MAA and DAA approach yielded excellent and similar early clinical outcomes. However, better patient-reported outcomes could be achieved by MAA THA. The MAA resulted in a safer approach associated with shorter operative times, less blood loss, lower Hb drop, fewer blood transfusions, and LFCN neuropraxia than DAA. A longer follow-up is needed to further examine differences between these procedures.

## Introduction

Primary total hip arthroplasty (THA) is generally considered one of the most successful surgeries in modern medicine, leading to pain relief, functional recovery, and improved quality of life [1]. Technological advancements matched with low revision rates have resulted in an increase in ever younger candidates undergoing THA [2, 3]. Patients seeking THA are not only interested in the resolution of pain and restoration of function but also a faster recovery [3–5].

Over recent years, THA performed through a minimally invasive approach, which provides earlier pain relief and functional recovery, has been widely used [6, 7]. The direct anterior approach (DAA) causes less trauma to soft tissue and shorter hospitalization compared to conventional approaches as it follows intermuscular interval and separates the anatomic interval between the tensor fasciae latae and the sartorius muscles to reach the hip joint [8]. On the other hand, the minimally anterolateral approach (MAA) separates the intermuscular plane between the tensor fasciae latae and the gluteus medius muscles. This is the modified Watson-Jones approach that was first reported by Bertin et al. [9] in 2004. Both MAA and DAA have been reported to be advantageous in THA, reducing postoperative pain and permitting quicker rehabilitation by preserving muscle insertions [10, 11].

However, to the best of our knowledge, there are limited comparative studies presenting early postoperative outcomes performed by a single surgeon via the MAA and DAA approaches. Therefore, we conducted this prospective study to further the understanding of the effects of MIS-THA on clinical and other parameters and assess the differences in early clinical, radiological, and patient-reported outcomes between the two minimally invasive approaches, i.e., MAA and DAA. We hypothesized that the MAA and DAA groups would provide similar early postoperative outcomes and that patients from both groups would recover early [12–14].

## Materials and methods

### Patient*s*

**Fig. 1:**
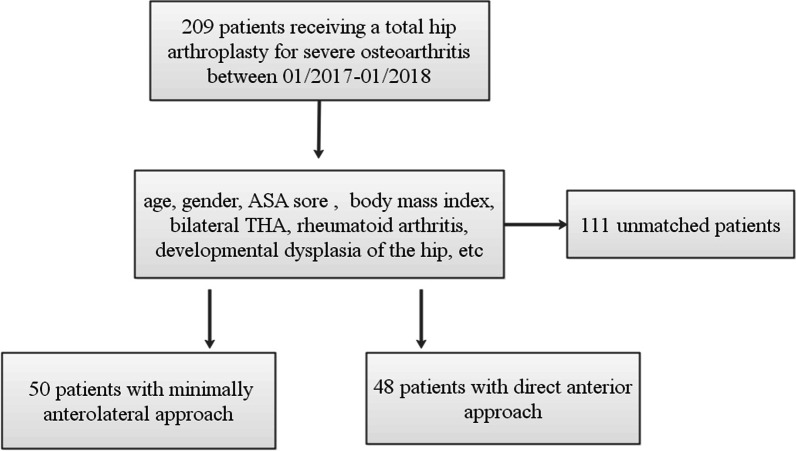
Patient selection chart

A total of 98 patients (98 hips) with hip osteoarthritis at the terminal stage who underwent primary unilateral cementless THA (Implant: Pinnacle Acetabular Cup System and Corail Hip System by DePuy, Johnson and Johnson, USA) between January 2017 and January 2018, were included in the study. The inclusion criteria were the following: patients with an underlying diagnosis of hip osteoarthritis; 20–80 years old; American Society of Anesthesiologists grade (ASA) ≤ 3; and a body mass index (BMI) < 30 kg/m^2^. The research met the diagnostic criteria of hip osteoarthritis provided by the Guideline of Diagnosis and Treatment of Osteoarthritis [15]. X-ray film showed that osteophyte was present in one hip. The exclusion criteria were those with a previous history of hip surgery, rheumatoid arthritis, avascular necrosis of the femoral head, neuromuscular disease, and deformity in limb joints other than the hip joint. Patients were randomized to MAA (*n* = 50) or DAA groups (*n* = 48), using computer-generated cards (Fig. 1).

### Operative techniques

All surgeries were performed by one senior surgeon, who started MAA and DAA in 2011 and had practiced more than 100 THAs via MAA and more than 200 THAs via DAA before participating in this study.

In group MAA, the patient was placed in a supine position on the fracture table, after which an oblique skin incision about 9–11 cm was made depending on the build of the patient, extending distally from 2 cm lateral and distal to the anterior superior iliac spine and ending at the top of the greater trochanter. The subcutaneous tissue and fascia were opened. The intermuscular interval between the anterior border of the gluteus medius and the tensor fasciae latae was performed by blunt dissection with the insertion of the index finger until the femoral neck was reached. The lateral circumflex artery crossing this interval was ligated, while tensor fascia latae, glutei medius, and minimus were not split or detached. The capsule was divided in a circular arc fashion along the base of the femoral neck and was preserved. The surgeon externally rotated the involved lower limb for the femur preparation, adduction was performed, and the fracture table was extended for 15° to provide a hyperextension position. An elevating retractor was inserted posterior to the greater trochanter to lift the femur for more extensive exposure. After insertion of implants and checking for stability and length, the hip capsule was sutured, no drainage tubes were placed, and the wound was sutured in a standard layered fashion.

In group DAA, the patient was placed on a fracture table in the supine position. An 8–12-cm-long skin incision was made about 3 cm lateral and distal to the anterior superior iliac spine and along the anterior margin of the tensor fasciae latae. The intermuscular space was bluntly opened between the sartorius muscles and tensor fasciae latae. The capsule was anteriorly exposed, and a femoral neck osteotomy was performed. The posterior capsule was partially released for femoral preparation, and the greater trochanter could be elevated with a retractor to provide sufficient exposure. The femoral components were inserted in the same manner as in group MAA with press-fit fixation. No redon drainage was intra-articularly placed, and routine closure was performed.

### Perioperative interventions

All participants received the same standardized preoperative and postoperative treatment, including pain management and rapid rehabilitation [16]. Also, all patients received prophylactic antibiotics and thromboprophylaxis. Physical therapy was initiated on the first postoperative day, and all patients were allowed to abandon the crutches for full weight-bearing tolerance, depending on the individual immediate postoperative recovery and clinical condition. Patients were discharged from the hospital after a minimum hospital stay of 2 days (range 2–6 days).

### Clinical evaluations

Patients' background data and surgical data, including operating time, intraoperative blood loss, length of skin incision, blood transfusion, and post-op hospital stay, were recorded. On a postoperative day 1, hemoglobin (Hb) was measured, and the number of blood transfusions was also recorded. (Blood transfusion was required when hemoglobin < 70 g/L.) Evaluations were conducted by two physical therapists employed at the following time points: preoperatively and postoperatively at 2, 6, and 12 weeks. Clinical results were evaluated using the ability to climb stairs and walk, 6-min walk test (6MWT) [17], visual analog scale (VAS, 1–10 point scale), the Forgotten Joint Scale (FJS-12), and Japanese Orthopedic Association (JOA) Hip scores. The FJS-12 is a self-administered questionnaire that estimates patients’ awareness of their knee or hip joint during activities of daily living, symbolizing a specific but very subjective patient-reported outcomes measure, with the eventual score ranging from 0 (worst) to 100 (best) [18]. JOA score has four subcategories: pain (Pain, 40 points), range of motion (ROM, 20 points), ability to walk (Gait, 20 points), and activities of daily living (ADL, 20 points); a high JOA score is indicative of better hip function [19]. In addition, the presence of any other complications after operating was recorded during follow-up.

### Radiographic evaluations

Preoperative, 24 h postoperative, 12 weeks postoperative, digitalized radiographs with an anteroposterior pelvis and cross-table lateral were routinely performed in all cases. Radiographs obtained at 12 weeks postoperative were examined to assess cup alignment (inclination angle, radiographic anteversion angle) and stem alignment (graded as valgus, neutral, or varus) [20]. Anatomical anteversion evaluated at 24 h postoperative computed tomography (CT) was converted into radiographic anteversion [21]. All radiographs were examined by two independent reviewers who were not directly involved with patient treatment and thus were blinded to all clinical information during the assessment of radiographic implant alignment.

### Statistical analysis

We compared clinical outcomes and implant alignment between MAA and DAA groups. A histogram was used to assess the normal distribution. Continuous scales were compared with the two-sample *t* test for outcomes with normal distribution or Wilcoxon’s rank-sum for outcomes with non-normal distribution; categorical variables were compared by Pearson’s *χ*^2^ test or Fisher’s exact probability test using SPSS version 20.0 (SPSS Inc., Chicago, IL). A *P* value < 0.05 was considered statistically significant.

## Results

### Clinical outcomes

Demographic data were compared between the MAA and DAA groups (Table 1). There were no significant differences in age, gender, BMI, operative side, ASA status, 6 MWT, preoperative VAS, or preoperative JOA hip score between MAA and DAA groups (*P* > 0.05). Surgical wounds, postoperative hospital stay, and postoperative VAS scores at all time points also did not significantly differ between the groups (*P* > 0.05) (Table 2).

**Table 1:** Demographic data on the patients

Variable	MAA	DAA	*P* value
Age (years)	62.44 ± 7.07	62.21 ± 8.22	0.881
Males/females (*n*)	17 (34.0%)/33(66.0%)	23 (47.9%)/25(52.1%)	0.161
BMI (kg/m^2^)	22.46 ± 4.23	22.96 ± 3.11	0.509
Operative side—right (*n*)	23 (46.0%)	25 (52.1%)	0.547
ASA score (I:II:III)	11/30/9	9/34/5	0.460
6 MWT (m)	289 ± 51.36	279.17 ± 57.05	0.372
Pre-op VAS (points)	7.32 ± 1.02	7.23 ± 1.12	0.675
Pre-op JOA (points)			
Pain	16.74 ± 4.58	16.73 ± 5.37	0.991
ROM	12.98 ± 2.99	12.21 ± 3.29	0.227
Gait	10.54 ± 3.45	11.02 ± 3.49	0.494
ADL	11.32 ± 3.63	10.35 ± 3.08	0.160
Total	51.58 ± 9.69	50.31 ± 9.77	0.521

**Table 2:** Surgical and immediate postoperative data

Variable	MAA	DAA	*P* value
Surgery time (min)	62.4 ± 9.05	71 ± 8.01	**< 0.001**
Incision (cm)	9.86 ± 0.99	10.04 ± 1.07	0.385
Blood loss (ml)	132.6 ± 43.31	159.23 ± 37.25	**0.002**
Hb drop (g/L)	29.56 ± 8.02	36.4 ± 7.12	**< 0.001**
Blood transfusion (*n*)	2 (4.0%)	9 (18.8%)	**0.021**
Post-op hospital stay (day)	4.6 ± 1.14	4.77 ± 1.17	0.467
VAS (points)			
1 day post‐op	4.2 ± 0.99	4.38 ± 0.82	0.343
3 day post‐op	2.82 ± 0.83	2.79 ± 0.8	0.863
7 day post‐op	2.24 ± 0.52	2.38 ± 0.57	0.222
Cup inclination (°)	41.96 ± 4.45	40.71 ± 4.09	0.151
Cup anteversion (°)	16.64 ± 5.63	15.92 ± 5.01	0.504
Femoral stem position (*n*)			0.963
Valgus	0 (0%)	0 (0%)	
Neutral	48 (96.0%)	45 (93.8%)	
Varus	2 (4.0%)	3 (6.2%)	

*P* value < 0.05 was shown in bold

**Fig. 2:**
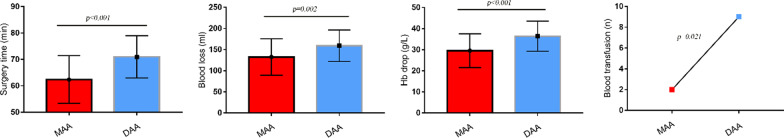
Surgical and immediate postoperative difference data: surgery time, blood loss, Hb drop, and the number of blood transfusions

The surgery time was significantly shorter in the MAA group than in the group DAA [(62.4 ± 9.05) min vs. (71 ± 8.01) min, *P* < 0.001]. The blood loss for MAA subjects was (132.6 ± 43.31) ml vs. (159.23 ± 37.25) ml for the DAA patients (*P* = 0.002). The Hb drop was also significantly lower on the first day postoperatively for MAA group (*P* < 0.001). Two (4.0%) participants and 9 (18.8%) participants in the MAA and DAA groups, respectively, required blood transfusions for anemia; the observed difference was statistically significant (*P* = 0.021) (Table 2 and Fig. 2).

**Fig. 3:**
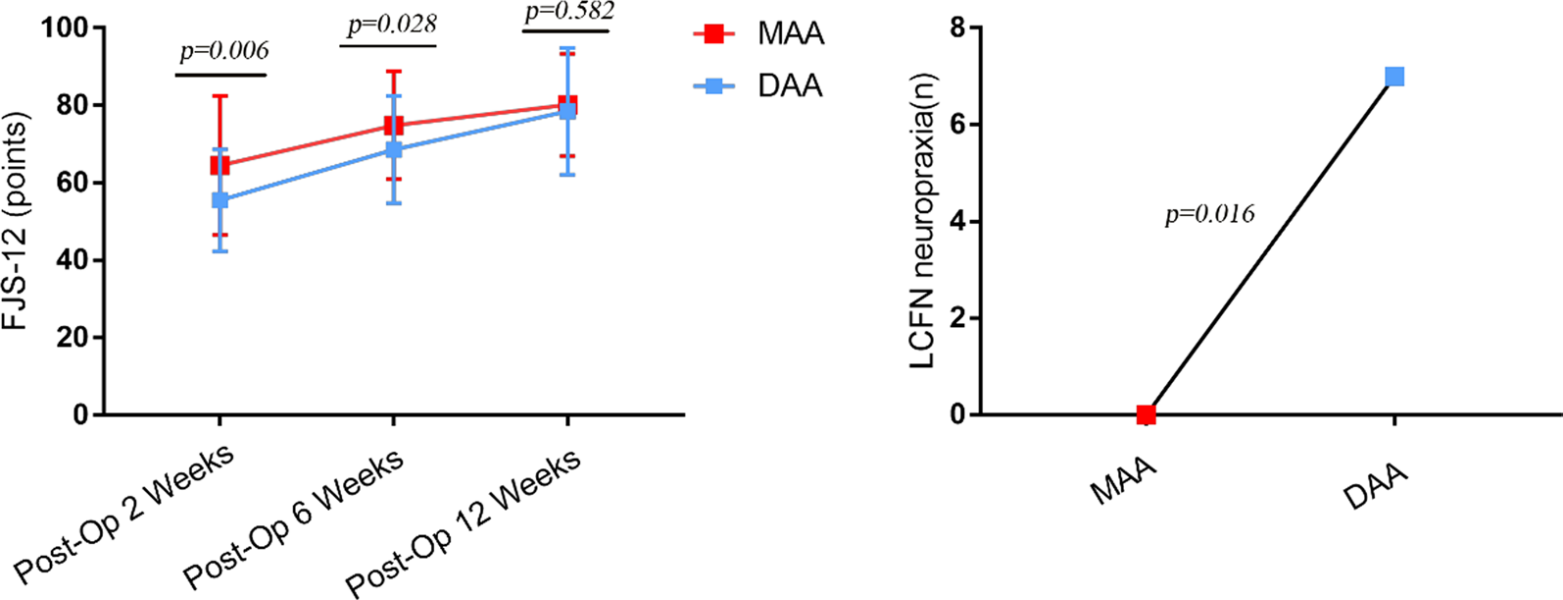
Postoperative outcomes and surgical complications difference data: FJS-12 and the number of LCFN neuropraxia

There were no significant differences in those from MAA group who were using stairs normally, walking unlimited, 6MWT, and wearing shoes and socks with ease at 2, 6, and 12 weeks postoperatively compared to the DAA group (*P* > 0.05). In the comparison of JOA scores in both groups 2 and 6 and 12 weeks after surgery, there were no statistically significant differences in any of the measured parameters (*P* > 0.05). In regards to patient-reported outcomes, the FJS-12 was significantly higher in the MAA group than in the group DAA at 2 (*P* = 0.006) and 6 weeks (*P* = 0.028) postoperatively (64.5 ± 17.96 vs. 55.52 ± 13.18, 74.9 ± 13.98 vs. 68.65 ± 13.84), respectively. There was no significant difference in the FJS-12 between the two groups at 12 weeks after surgery (*P* = 0.582) (Table 3 and Fig. 3).

**Table3:** Postoperative outcomes data

Variable	2 weeks			6 weeks			12 weeks		
	MAA	DAA	*P* value	MAA	DAA	*P* value	MAA	DAA	*P* value
VAS (points)	2.16 ± 1.2	2.29 ± 1.32	0.607	1.86 ± 1.16	1.85 ± 1.24	0.981	1.4 ± 1.14	1.13 ± 0.84	0.180
Stairs normally (*n*)	16 (32.0%)	14 (29.2%)	0.761	24 (48.0%)	26 (54.2%)	0.542	42 (84.0%)	36 (75.0%)	0.269
Distance unlimited (*n*)	25 (50.0%)	20 (41.7%)	0.408	38 (76.0%)	39 (81.3%)	0.527	42 (84.0%)	42 (87.5%)	0.621
Shoes and socks with Ease (*n*)	21 (42.0%)	24 (50.0%)	0.427	36 (72.0%)	32 (66.7%)	0.567	42 (84.0%)	43 (89.6%)	0.415
6MWT(m)	225 ± 87.74	238.54 ± 86.17	0.443	346.6 ± 86.26	332.29 ± 72.24	0.377	410.6 ± 82.69	425.83 ± 86.44	0.375
FJS-12 (points)	64.5 ± 17.96	55.52 ± 13.18	**0.006**	74.9 ± 13.98	68.65 ± 13.84	**0.028**	80.2 ± 13.21	78.54 ± 16.44	0.582
JOA (points)									
Pain	29.2 ± 4.5	30.81 ± 5.17	0.102	36.76 ± 2.54	36.02 ± 2.42	0.143	38.98 ± 1.8	38.44 ± 2.61	0.232
ROM	13.88 ± 3.27	14.5 ± 3.59	0.373	16.28 ± 1.33	16.52 ± 2.41	0.539	16.96 ± 1.73	17.38 ± 1.95	0.267
Gait	13.6 ± 2.96	12.94 ± 3.48	0.312	14.1 ± 3.87	15.33 ± 3.22	0.090	18.06 ± 1.75	17.42 ± 2.56	0.148
ADL	13.9 ± 3.02	14.44 ± 2.7	0.356	15.04 ± 3.52	15.88 ± 3.55	0.245	16.76 ± 1.51	17.25 ± 1.64	0.127
Total	70.58 ± 7.49	72.69 ± 9.09	0.213	82.52 ± 7.62	84.33 ± 6.77	0.217	90.76 ± 3.16	90.48 ± 6.97	0.797

*P* value < 0.05 was shown in bold

### Radiological outcomes

There were no statistically significant differences in film positioning of implants between the MAA and DAA groups (*P* > 0.05; Table 2). On 12-week radiographs, the MAA group had an average cup inclination of 41.96° ± 4.45 compared to an average cup inclination of 40.71° ± 4.09 in the DAA group. Acetabular anteversion was 16.64° ± 5.63 in the MAA and 15.92° ± 5.01 in the DAA group. No cups in either the MAA or DAA groups presented with a sign of migration at 12 weeks. The MAA group had 2 (4.0%) stem implanted compared to 3 (6.2%) in the DAA group, but this difference was not statistically significant (*P* = 0.963). Femoral implants that subsided stabilized without radiological evidence of subsidence of > 3 mm in both groups at 12 weeks.

### Complications

Surgical complications are described in Table 4. One fracture was identified in each group and managed conservatively. In the MAA group, an asymptomatic partial fracture of the greater trochanter (Vancouver classification AG) was identified 2 weeks postoperatively. It occurred due to a fall that did not require surgical fixation and was conservatively managed with bed rest and touch-weight-bearing, completely healing after 6 months of follow-up. In the DAA group, one case had an intraoperative femoral perforation on broaching, and full weight-bearing was prohibited for 6 weeks. The minor complication profile was significantly inclined to the DAA group as a result of the high incidence of lateral femoral cutaneous nerve (LFCN) neuropraxia. The leading symptom was dysesthesia of the lateral thigh. The incidence of LFCN neuropraxia after surgery was 7(14.6%) in the DAA group and 0 in the group MAA; the observed difference was statistically significant (*P* = 0.016). No complications of calcar fracture or postoperative dislocation were discovered at 12 weeks postoperatively (Table 4 and Fig. 3).

**Table 4:** Surgical complications

Complication	MAA (*n*)	DAA (*n*)	*P* value
Superficial infection (hips)	0	0	1.00
Deep infection	0	0	1.00
DVT	0	0	1.00
Calcar fracture	0	0	1.00
Major trochanteric fracture	1 (2%)	1 (2.1%)	1.00
LCFN neuropraxia	0	7(14.6%)	**0.016**
Nerve palsy	0	0	1.00
Postoperative dislocation	0	0	1.00
Postoperative femoral fracture	0	0	1.00
Cup migration	0	0	1.00
Stem subsidence > 3 mm (EBRA/FCA)	0	0	1.00
Thigh pain	0	0	1.00
Bursitis	0	0	1.00

*P* value < 0.05 was shown in bold

## Discussion

Both DAA and the MAA, minimally invasive approaches in total hip arthroplasty (THA), have been associated with less muscle damage, less perioperative pain, and rapid recovery [12]. DAA and MAA allow for exposure of the hip joint between inter-muscular planes with a lower risk of dislocation and without increasing the risk of early revision [22]. However, there have been few comparative studies about early postoperative outcomes between the DAA and MAA. In the present study, we observed similar primary early clinical outcomes following MAA and DAA THA, i.e., the ability to climb stairs and walk, 6MWT, VAS pain, and JOA scores. Postoperative hospital stay was also similar in both groups. The similar clinical outcomes suggested that pain relief and functional recovery over the total hip did not significantly differ among groups, which may be due to the reduced invasiveness of both surgical approaches; this is consistent with other research findings [12]. Despite the overall similarity of the MAA and DAA outcomes, some significant differences were confirmed in other domains. Two and 6 weeks postoperative FJS-12 scores were significantly better in the MAA group, which suggested that better patient-reported outcomes could be achieved by MAA THA. Nevertheless, the present study revealed that 12 weeks after surgery, the FJS-12 score was similar between the MAA and DAA groups. Differences in favor of the MAA also included shorter operative times, less blood loss, lower Hb drop, fewer blood transfusions, and LFCN neuropraxia. Implant alignment is essential for successful longtime outcomes [23]. Luger et al. [24] evaluated differences in planning adherence between the MAA and the DAA in cementless short stem THA, showing no statistically significant difference in offset reconstruction, leg length, or implant positioning between groups. Similarly, Kawarai et al. [22] conducted a study to clarify the difference in implant alignment between the MAA and DAA approach. They found that both cup and stem alignments improved in both groups. Yet, they found that the frequency of the safe zone and stem alignment in the sagittal plane was more favorable in the MAA group than in the DAA group. The difference with the MAA procedure was lateralization of the proximal femur through medialization of the tensor fasciae latae muscle, as lateralization of the proximal femur can avoid the prominence of the iliac crest along with the linear extension of the femoral canal axis. Therefore, it can reduce operation time and blood loss on the femoral side [22].

The DAA and MAA approach is an internervous or intermuscular approach, connected with faster rehabilitation and faster recovery. These characteristics are beneficial for total blood loss and transfusion rate [12, 25]. However, the MAA approach showed better results in this study. The operation steps associated with the MAA partly explained the shorter surgical times with less blood loss, lower Hb drop, and fewer blood transfusions. The decreased blood loss, which was generally on the femoral side, was related to an easier visualization of posterolateral capsular bleeders associated with the MAA. When THA was performed via the DAA, a release of a portion of the posterolateral capsule and piriformis was at times necessary during the elevation of the proximal femur. Surgeon experience also was also an important factor, where a surgeon in the present study had over 100 MAA cases versus 200 DAA cases. Still, the longer surgical time and greater blood loss were not compensated by better pain relief, faster function improvement, and earlier discharge in the DAA group.

This study presents favorable implant alignments in THA with both minimally invasive techniques. The MAA and DAA groups showed similar alignments of both cup and stem. Component positioning has a marked impact on the function and duration of THA [26–28]. The MAA and DAA surgical approaches can expose the hip joint intermuscular and internervous interval with a lower chance of dislocation and without higher revision risk [29, 30]. Kawarai et al. [22] reported that cup anteversion in DAA is nearly 4°larger than MAA in the supine position, which is inconsistent with our results. The larger anteversion in DAA could be due to the difference in rotation and tilt angles of the pelvis in the supine position. This could lead to flexor muscles and femoral weight. During the cup procedure, the ipsilateral pelvis could be posteriorly retracted. According to Kobayashi et al. [31], it is somewhat challenging to insert the femoral stem in the neutral position via DAA due to the exposure of the proximal femur. Inconsistent with Kobayashi et al. reports, the proximal femur could be adequately exposed to find the entry point in the DAA group. There was no statistically significant difference in stem implant alignment compared with the MAA group. Adequate exposure was obtained by a longer surgical time to manage the femur side.

Our study's risk of minor surgical complications was lower in the MAA group due to the 14% incidence of LFCN neuropraxia in the DAA group. LFCN neuropraxia was a typical complication of DAA. The DAA for THA is the only true internervous approach to the hip that can leave abductors and posterior soft tissue envelope intact. The LFCN is endangered if the incision is too close to the anterior superior iliac spine, with a distal extension of the incision (small distal branches), with hyperextension of the hip, with dissection of the medial subcutaneous fat pad, with aggressive or prolonged retraction of the rectus femoris during component implantation, and with involvement in tissue scarring [32]. It is hoped that technical improvement, such as moving the incision more laterally, would help reduce the incidence following this approach [32]. Ozaki et al. [33] demonstrated that LFCN neuropraxia mainly resolves spontaneously overtime after THA. The incidence in the present study was similar to the previously reported range in the literature [3, 34, 35]. There were no connections between LFCN neuropraxia and 6MWT or JOA scores; however, higher FJS-12 scores were found in MAA group. This was supported by Ozaki et al. [33].

The present study has some limitations. First, this was an early evaluation of only 12 weeks after surgery. A longer follow-up is needed to compare the effects of the MAA and DAA methods on LFCN neuropraxia, FJS-12 scores, and implant alignment after THA. Second, the study could not be fully blinded due to the visible differences in the surgical incisions. All radiographs reviews were blinded by two independent reviewers. In addition, only a cementless stem was utilized. Generally, a narrower exposure of the proximal femur is the only requirement for a cementless stem to prepare the femur compared to a cemented stem [36, 37]. In this manner, minimally invasive surgery becomes less technically demanding and generates an advantage for the cementless stem.

## Conclusion

Both the MAA and DAA approaches yielded excellent and similar early clinical outcomes. However, the MAA approach may be safer for weak patients. Also, moving the incision more laterally in the DAA approach may provide a better patient's self-awareness of their hip joint during activities of daily living for reduced incidence of LFCN neuropraxia.

## Data Availability

Not applicable.

## Abbreviations

THA: Total hip arthroplasty
MIS-ALA: Minimally anterolateral approach
DAA: Direct anterior approach
6MWT: 6-Minute walk test distances
FJS-12: Forgotten Joint Scale
JOA: Japanese Orthopaedic Association
LFCN: Lateral femoral cutaneous nerve
ASA: American Society of Anaesthesiologists grade
BMI: Body mass index
VAS: Visual analog scale
CT: Computed tomography
